# Primary gross tumor volume is prognostic and suggests treatment in upper esophageal cancer

**DOI:** 10.1186/s12885-021-08838-w

**Published:** 2021-10-21

**Authors:** Yuanmei Chen, Qiuyuan Huang, Junqiang Chen, Yu Lin, Xinyi Huang, Qifeng Wang, Yong Yang, Bijuan Chen, Yuling Ye, Binglin Zheng, Rong Qi, Yushan Chen, Yuanji Xu

**Affiliations:** 1 Department of Thoracic Surgery, Fujian Medical University Cancer Hospital, Fujian Cancer Hospital, Fuzhou, Fujian China; 2 Department of Radiation Oncology, Fujian Medical University Cancer Hospital, Fujian Cancer Hospital, No. 420, Fuma Road, Fuzhou, 350014 China; 3 Department of Radiation Oncology, Sichuan Cancer Hospital Institute, Sichuan Cancer Center, School of Medicine, University of Electronic Science and Technology of China, Chengdu, China; 4 Department of Radiation Oncology, National Cancer Center/National Clinical Research Center for Cancer/Cancer Hospital, Chinese Academy of Medical Sciences and Peking Union Medical College, Beijing, China

**Keywords:** Upper ESCC, GTV-p, Prognosis, Treatment

## Abstract

**Background:**

To aid clinicians strategizing treatment for upper esophageal squamous cell carcinoma (ESCC), this retrospective study investigated associations between primary gross tumor volume (GTVp) and prognosis in patients given surgical resection, radiotherapy, or both resection and radiotherapy.

**Methods:**

The population comprised 568 patients with upper ESCC given definitive treatment, including 238, 216, and 114 who underwent surgery, radiotherapy, or combined radiotherapy and surgery. GTVp as a continuous variable was entered into the multivariate Cox model using penalized splines (P-splines) to determine the optimal cutoff value. Propensity score matching (PSM) was used to adjust imbalanced characteristics among the treatment groups.

**Results:**

P-spline regression revealed a dependence of patient outcomes on GTVp, with 30 cm^3^ being an optimal cut-off for differences in overall and progression-free survival (OS, PFS). GTVp ≥30 cm^3^ was a negative independent prognostic factor for OS and PFS. PSM analyses confirmed the prognostic value of GTVp. For GTVp < 30 cm^3^, no significant survival differences were observed among the 3 treatments. For GTVp ≥30 cm^3^, the worst 5-year OS rate was experienced by those given surgery. The 5-year PFS rate of patients given combined radiotherapy and surgery was significantly better than that of patients given radiotherapy. The surgical complications of patients given the combined treatment were comparable to those who received surgery, but radiation side effects were significantly lower.

**Conclusion:**

GTVp is prognostic for OS and PFS in upper ESCC. For patients with GTVp ≥30 cm^3^, radiotherapy plus surgery was more effective than either treatment alone.

**Supplementary Information:**

The online version contains supplementary material available at 10.1186/s12885-021-08838-w.

## Background

Esophageal cancer (EC) ranks seventh in cancer incidence and sixth in mortality worldwide [1]. Surgery is usually the cornerstone for curative treatment. Yet, while cancer of the upper third of the esophagus at the cervical and upper thoracic region is relatively rare (accounting for no more than 10% all cases) [2], radical esophagectomy is often precluded. This is because upper third EC, especially cervical, is histologically almost entirely squamous cell, with a high risk of local invasion of adjacent anatomical structures.

The guideline of the National Comprehensive Cancer Network (NCCN) provides no consensus regarding the optimal treatment of upper EC, although definitive radiotherapy is recommended for cervical EC [3]. Unfortunately, upper third EC has a worse prognosis than middle or lower third tumors, with an estimated 5-year overall survival rate of 33% [4].

Prognosis for EC has traditionally relied on tumor-node-metastasis (TNM) staging [4, 5], but there is accumulating evidence that primary gross tumor volume (GTVp) is particularly prognostic and may guide clinical treatment [6]. With advances in diagnostic imaging and therapeutic technology, GTVp has the potential to become a powerful predictor of patient survival. In 2006, Créhange et al. [7] reported that patients with EC had a worse prognosis if the GTVp was 100 cm^3^ or larger. In 2015, it was demonstrated that primary GTVp was not only a significant predictor of improved survival, but also more powerful than TNM staging as a prognostic factor [8]. In 2019, Lv et al. [9] showed that relative tumor volume was an independent prognostic factor in non-resectable esophageal squamous cell carcinoma (ESCC), and GTV for lymph nodes was an independent indicator of distant metastasis-free survival. In the same year, Chen et al. [10] also reported that GTVp was an independent factor that affected overall survival in ESCC, which could be combined with N stage to improve prediction. Hence, GTVp is an important prognostic factor that may provide new insight for personalized treatment strategies in EC.

Due to the rarity of upper ESCC, prognostic factors remain unclear [11]. Based on the above studies, it is speculated that GTVp may also be prognostic for survival in upper ESCC, and help guide treatment. The current treatment modalities for upper ESCC are mainly radiotherapy, surgery, or radiotherapy in combination with surgery. In addition, some studies have shown that concurrent chemoradiotherapy was effective against upper third ESCC [11, 12], or that upper third EC was especially sensitive to preoperative chemoradiotherapy, which significantly improved survival [13]. Surgery has even been demonstrated recently as better than radiotherapy for therapeutic effect in upper third ESCC [14]. Nevertheless, GTVp was not examined in such studies, and further investigation is warranted concerning optimal therapeutic schedules based on GTVp for upper ESCC.

The current study explored the prognostic value of GTVp in upper ESCC, and the value of GTVp for strategizing individualized treatment. As such, this study provides a clinical reference for individualized treatment of patients with upper ESCC.

## Materials and methods

### Patients and pretreatment assessment

This retrospective study was performed with the approval of Fujian Medical University Cancer Hospital Institutional Board Review (No. SQ2020–063-01).

For inclusion in the present analysis, patients fulfilled the following criteria: given no prior treatment; with pathologically proven ESCC without distant metastases; the tumor located at the upper thoracic region (as defined by the NCCN guideline [3]); and designated ≤3 by Eastern Cooperative Oncology Group scoring. The pretreatment assessment of all patients was performed according to our institutional protocol [10]. Clinical TNM staging was in accordance with the eighth edition of the Union for International Cancer Control/American Joint Committee on Cancer (UICC/AJCC) staging criteria for EC. Potential subjects were excluded if they had recurrent disease; secondary malignancies; or any tumor located in the cervical, middle, or lower third thoracic esophagus.

Finally, 568 patients with upper ESCC were enrolled in this analysis. Patients were apportioned to the following groups, based on treatment given: definitive radiotherapy/concurrent chemotherapy (RT); radical surgery (S); or pre- or postoperative radiotherapy as well as radical surgery (S + RT). The R, S, and R + S groups consisted of 238, 216, and 114 patients, respectively. Patients in each group may or may not have also received pre- or postoperative chemotherapy, and neoadjuvant or adjuvant chemotherapy of radiotherapy.

### GTVp measurement

Chest computed tomography images before treatment were transmitted to a 3D treatment-planning system. Primary target lesions, excluding lymph nodes, were independently outlined by 2 thoracic radiotherapists, each therapist with at least 10 years of clinical practice in the treatment of EC. Any discrepancies were resolved by consensus. The 3D images were automatically reestablished and GTVp measurements were automatically computed by the system.

### Treatments

Surgical treatment consisted of radical resection of local tumor and regional lymph nodes, in accordance with institutional procedures described previously [15]. Radiotherapy technology included 3-dimensional conformal radiation therapy or intensity modulated radiation therapy. The definitive [4], pre- [10], and postoperative [16] radiotherapy treatments, including the targets, target dose, and dose limitations to organs at risk have been described as cited. The median radiation dose to the targets for definitive, pre-, and postoperative radiotherapy were, respectively, 61.5 (range, 50.0 to 67.2), 40.0 (36.0 to 50.0), and 50.0 (40.0 to 63.0) Gy with conventional fractions.

Chemotherapy treatments were platinum-based, and administered to 370 (65.1%) of the patients, of whom 113 (30.5%), 163 (44.1%), and 94 (25.4%) were in the S, RT, and S + RT groups, respectively.

### Evaluation of treatment adverse effects

The main observational indexes of complications associated with radical surgery were: pneumonia, anastomotic leakage, wound infection, chylothorax, respiratory failure, anastomotic stenosis, and cardiac insufficiency. The main observational indexes of acute toxicities of radiotherapy were acute radiation-induced esophagitis and radiation-induced pneumonitis, which were evaluated based on the criteria of the Radiation Therapy Oncology Group [17].

### Surveillance

The surveillance schedule for patients was performed as in our previous study [10]. In short, patients were followed-up every 3 months for the first 2 years; every 6 months for years 3 to 5; and annually thereafter. The final follow-up date was 19 December 2019. The median surveillance time was 41.5 months. The primary endpoint was overall survival (OS), which was calculated from the start of diagnosis to the date of death or last follow-up. The secondary endpoint was progression-free survival (PFS), defined as the date of diagnosis to the date of death, local and/or regional relapse, distant metastasis, or last follow-up.

### Statistical analyses

The optimal cut-off of GTVp indicating a survival difference was evaluated using a multivariate Cox proportional hazards regression model, with penalized spline (P-spline) regression, which enabled a nonlinear association between GTVp and OS or PFS. P-spline was performed using the smoothHR package in R, version 3.5.2 (http://www.r-project.org/) [18]. Other data were analyzed using SPSS, version 24.0 (IBM, Armonk, NY, USA).

The clinical features and adverse effects of treatment of the different subgroups were compared with Pearson’s chi-squared or Fisher’s exact tests. OS and PFS rates were calculated using the Kaplan-Meier method, and subgroups were compared with the log-rank test. Multivariate analyses of clinical features were performed to determine independent prognostic factors for OS and PFS, via Cox proportional hazards regression. To adjust selection and confounding bias, the baseline characteristics for comparing different subgroups were compensated by using propensity score matching (PSM) analyses. The unbalanced factors were modulated by PSM with a match tolerance of 0.1. A 2-sided probability value *P* ≤ 0.05 indicated a statistically significant difference.

## Results

### Patient characteristics and treatment failure patterns

Among the 568 patients, there were 397 and 177 men and women, respectively (ratio 2.2:1), with a median age of 60 years (range, 38 to 90 y). There were 238, 216, and 114 in the RT, S, and RT + S treatment groups. Additional clinical details are summarized in Table 1, including clinical T (cT), clinical N (cN), and clinical TNM (cTNM) stages; lymph node metastasis (LNM); tumor length; and chemotherapy.

**Table 1:** Patient Characteristics by Treatment Group

Characteristics	Total	S	RT	S + RT	*P*
n(%)	568(100)	238(41.9)	216(38.0)	114(20.1)	
Gender					<0.001
Male	397(69.9)	152 (63.9)	143 (66.2)	96(84.2)	
Female	177(31.2)	86 (36.4)	73 (33.8)	18(15.8)	
age (year)					0.002
<60	276(48.6)	118(49.6)	88(40.7)	70(61.4)	
≥ 60	292(51.4)	120(50.4)	128(59.3)	44(38.6)	
LNM					<0.001
No	252(44.4)	135(56.7)	67(31.0)	50(43.9)	
Yes	316(55.6)	103(43.3)	149(69.0)	64(56.1)	
cT stage					<0.001
T0–2	127(22.4)	81(34.0)	20(9.3)	26(22.8)	
T3	247(43.5)	121(50.8)	65(30.1)	61(53.5)	
T4	194(34.2)	36(15.1)	131(60.6)	27(23.7)	
cN stage					<0.001
N0	285(50.2)	153(64.3)	67(31.0)	65(57.0)	
N1	185(32.6)	61(25.6)	91(42.1)	33(28.9)	
N2–3	99(17.3)	24(10.1)	58(26.9)	16(14.0)	
cTNM stage					
I-II	261(46.0)	157(66.0)	50(23.1)	54(47.4)	<0.001
III	117(20.6)	44(18.5)	41(19.0)	32(28.1)	
IV	190(33.5)	37(15.5)	125(57.9)	28(24.6)	
Tumor length					<0.001
≤ 5 cm	363(63.9)	180(75.6)	119(55.1)	64(56.1)	
>5 cm	205(36.1)	58(24.4)	97(44.9)	50(43.9)	
Chemotherapy					<0.001
Yes	370(65.1)	113(47.5)	163(75.5)	94(82.5)	
No	198(34.9)	125(52.5)	53(24.5)	20(17.5)	

For the entire cohort, the 5-year OS and PFS rates were 44.6 and 40.2%, respectively. The 5-year OS rates of the S, RT, and RT + S groups were 51.7, 34.2, and 51.0%; the 5-year PFS rates were 46.3, 29.0, and 49.6%. Ultimately, 319 patients died of the following: 105 of distant metastasis; 134 of local or regional relapse; and 11 and 69 of other medical or unknown causes. There were 148 patients who experienced tumor relapse (43, 86, and 19 of local, regional, or combined relapse). Overall, 113 patients developed distant metastasis.

### Volume-dependent effect of GTVp on OS and PFS

**Fig. 1:**
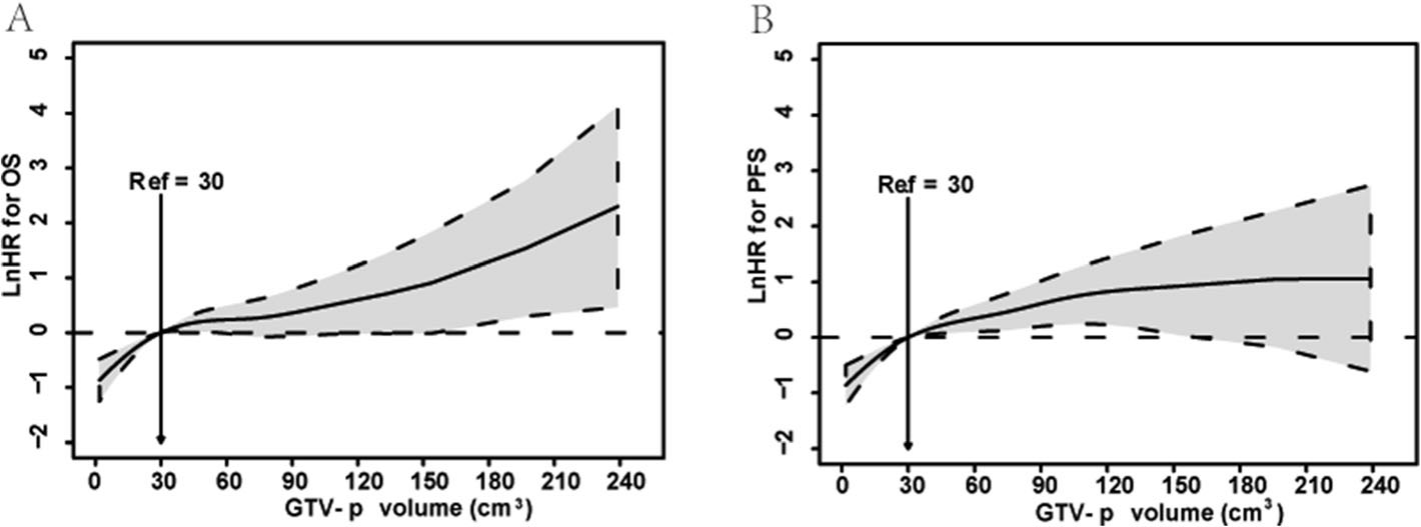
Optimal cutoff of GTV-p in 568 patients with upper ESCC. The effects of GTV-p on (**A**) OS and (**B**) PFS are modeled with a P-spline expansion, with GTV-p as a continuous variable. Estimated logarithm HRs (solid lines) with 95% confidence intervals (shaded area) for the association of GTV-p with OS or PFS according to the degrees of freedom in multivariate additive Cox in smoothHR - Optimal extended Cox - type additive hazard regression unadjusted model

To quantify the effect and optimal cutoff value of GTVp on OS and PFS, a multivariate Cox regression analysis was conducted using P-splines in smoothHR of the R software. The model indicated that the risk (InHR) of abbreviated OS and PFS increased sharply when GTVp was more than 30 cm^3^ (Fig. 1A, B). These findings confirmed a GTVp-dependent effect, and a GTVp of 30 cm^3^ was an optimal cut-off volume for survival difference. Consequently, all patients could be stratified into 2 subgroups based on a GTVp of less or greater than 30 cm^3^.

### Prognostic value of GTVp

**Fig. 2:**
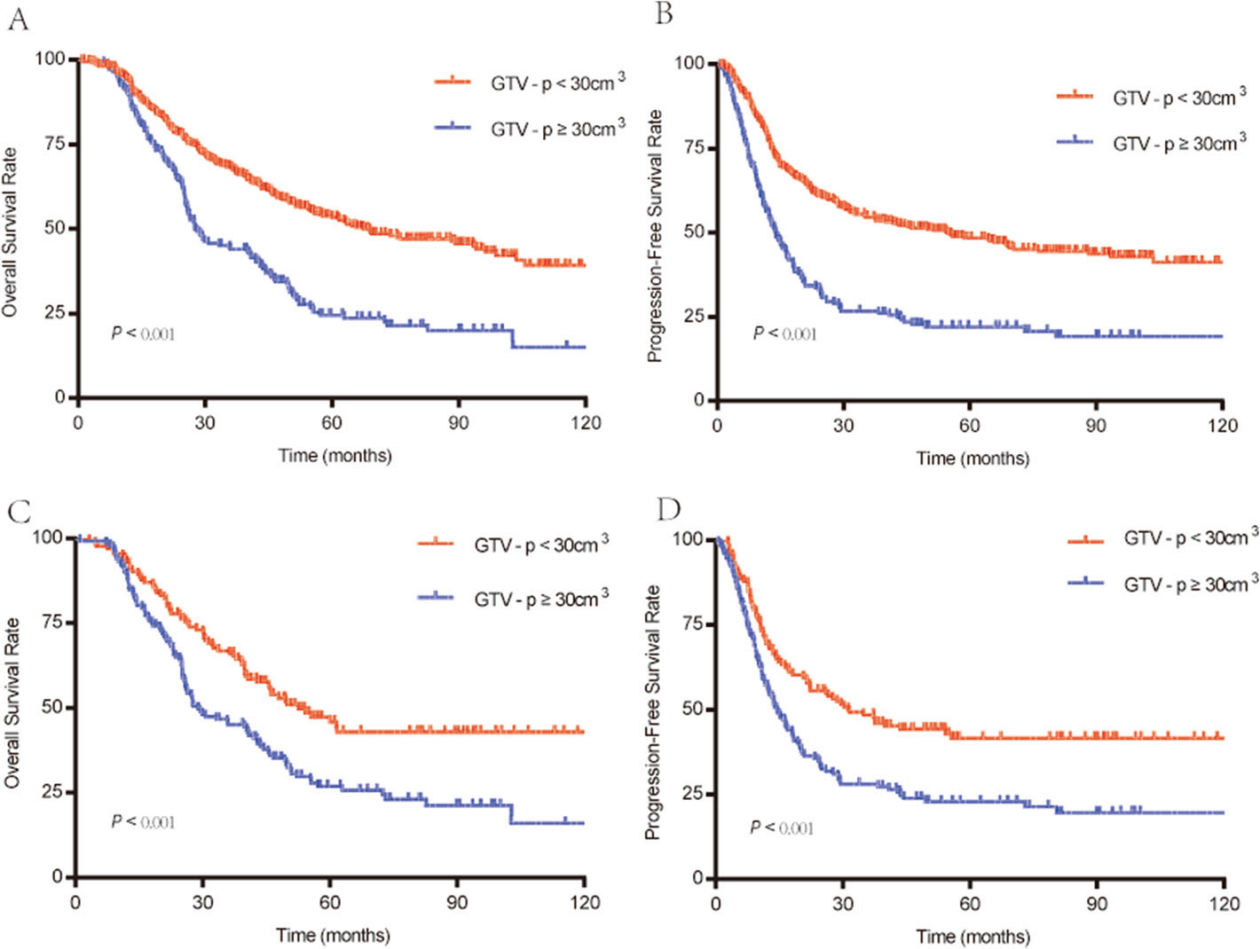
Overall survival rates of patients with GTV-*p* < 30 cm^3^ or GTV-*p* ≥ 30 cm^3^. Without PSM: (**A**) OS; (**B**) PFS. With PSM: (**C**) OS; (**D**) PFS

Overall, the average GTVp was 27.30 (1.85 to 238.99) cm^3^; specifically, 396 and 172 patients had, respectively, GTVp < 30 cm^3^ and ≥ 30 cm^3^. The prognostic value of GTVp was determined via univariate and multivariate analyses. The univariate analyses showed that patients with GTVp ≥30 cm^3^ had markedly poorer 5-year OS and PFS than did those with GTVp < 30 cm^3^ (Fig. 2A, B). Other prognostic factors for OS and PFS were: gender; cT, cN, and cTNM stage; LNM; and tumor length (Table 2). The multivariate Cox regression analysis confirmed the following as independent prognostic factors for OS and PFS: GTVp, gender, cT stage, and LNM. In addition, N stage was an independent prognostic indicator for PFS.

**Table 2:** Univariate and multivariate analyses of factors influencing OS and PFS

	OS		PFS		OS		PFS	
Characteristics	95% CI	*P*	95% CI	*P*	HR 95% CI	*P*	HR 95% CI	*P*
Age	0.835 ~ 1.297	0.721	0.780 ~ 1.195	0.748				
Gender	0.494 ~ 0.820	<0.001	0.543 ~ 0.883	0.003	0.676 0.522 ~ 0.875	0.003	0.775 0.606 ~ 0.990	0.041
GTV-p	1.625 ~ 2.550	<0.001	1.738 ~ 2.693	<0.001	1.682 1.329 ~ 2.129	<0.001	1.673 1.321 ~ 2.118	<0.001
LNM	1.615 ~ 2.581	<0.001	1.671 ~ 2.623	<0.001	1.803 1.419 ~ 2.292	<0.001	1.603 1.246 ~ 2.062	<0.001
cT stage	1.269 ~ 1.726	<0.001	1.309 ~ 1.762	<0.001	1.245 1.059 ~ 1.464	0.008	1.214 1.037 ~ 1.422	0.016
cN stage	1.313 ~ 1.732	<0.001	1.438 ~ 1.889	<0.001			1.264 1.077 ~ 1.483	0.004
cTNM stage	1.129 ~ 1.333	<0.001	1.156 ~ 1.357	<0.001				
Tumor length	1.177 ~ 1.839	0.001	1.186 ~ 1.829	<0.001				

Various clinical baseline characteristics were significantly different between the patients with GTVp < 30 cm^3^ and ≥ 30 cm^3^ (Supplemental Table 1), and PSM was employed to calibrate the potential indication bias between the 2 groups. After matching, the only significantly different clinical features were gender and tumor length, and 132 patients were matched in each group; GTVp remained a prognostic risk for OS and PFS (Fig. 2C, D). Taken together, these findings suggest that GTVp can independently predict OS and PFS.

### Prognostic value of treatment modalities stratified by GTVp

**Fig. 3:**
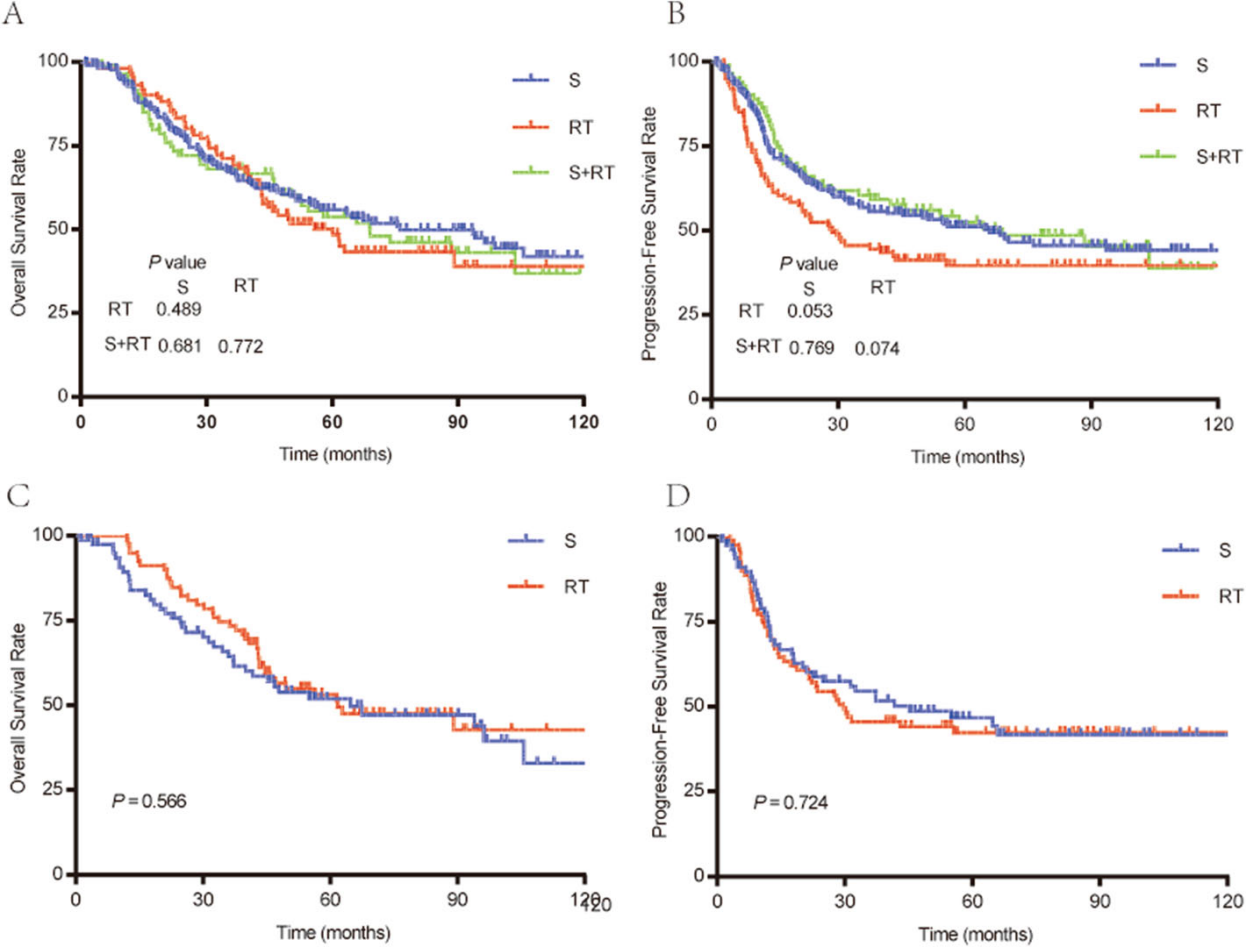
Survival rates of patients with GTV-p < 30 cm^3^. Without PSM among S, RT, and S + RT groups: (**A**) OS. (**B**) PFS. With PSM between S group and RT group: (**C**) OS. (**D**) PFS

As the GTVp of the 3 treatment groups differed so greatly, the possibility of selection bias was considered (Supplemental Table 1). Hence, it was necessary to explore the prognostic effect of treatment, stratified by GTVp. For GTVp < 30 cm^3^, the OS curves of the RT, S, and S + RT groups appeared to be identical and did not differ significantly (Fig. 3A). The PFS curves of the 3 groups were comparable, expect with a border line difference between the RT and S groups (*P* = 0.053; Fig. 3B).

The PSM analysis revealed no marked difference among the various clinical features of the 79 patients in the RT group and the 79 patients in the S group (Supplemental Table 2). It was observed that the OS and PFS curves of the RT and S groups overlapped, respectively (all *P* > 0.05; Fig. 3C, D).

**Fig. 4:**
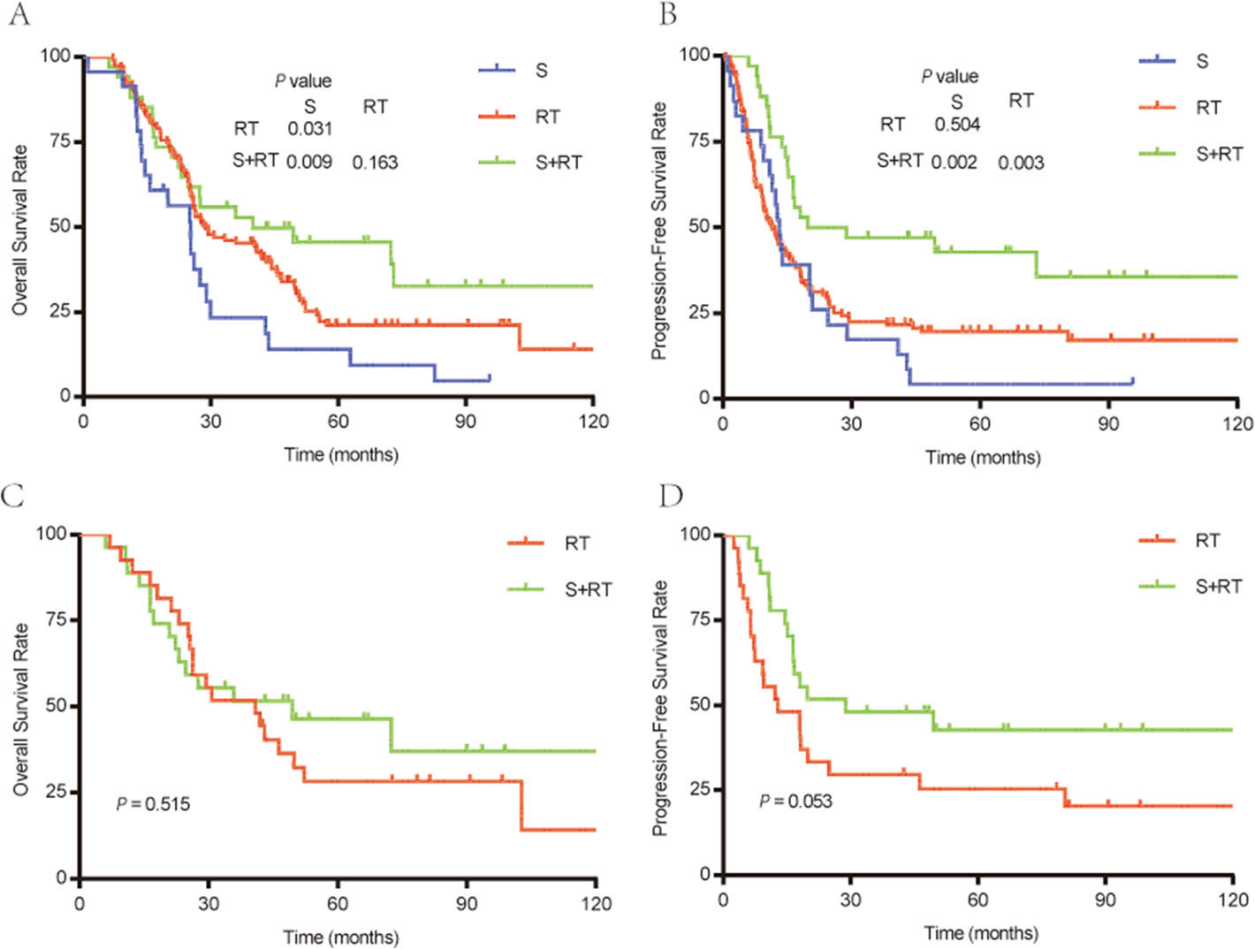
Survival rates of patients with GTV-p ≥ 30 cm^3^. Without PSM among S, RT, and S + RT groups: (**A**) OS. (**B**) PFS. With PSM between R group and R + S group: (**C**) OS. (**D**) PFS

For GTVp ≥30 cm^3^, the OS curves of the S + RT and RT groups were significantly better than that of the S group; the 5-year OS rates of the S + RT and RT groups were 45.4 and 21.1%, respectively, while that of the S group was 14.1% (*P* = 0.009 and 0.031; Fig. 4A). Although the 5-year OS rate of the S + RT group was better than that of the RT, the difference did not reach statistical difference (*P* = 0.169). In addition, the S + RT group showed markedly better distinction of PFS curves compared with the RT and S groups with the 5-year PFS being 42.8% cf. 19.7 and 42.8% cf. 14.3% (*P* = 0.003 and *P* = 0.002, respectively), whereas the PFS curves of the RT and S groups almost overlapped (*P* = 0.504; Fig. 4A, B). The PFS curve of the S + RT group was significantly better than that of the RT or S groups; the 5-year PFS rates of the S + RT group was 42.8%, whilst that of the RT and S groups were 21.1 and 14.1% (*P* = 0.003 and 0.002). The PFS curves of the RT and S groups almost overlapped (*P* = 0.504, Fig. 4A, B).

To compensate for the unbalanced baseline characteristics, 27 patients in the S + RT group and 27 in the RT group were selected for comparison of their PFS (Supplemental Table 3). The PFS of the 2 groups remained statistically similar (Fig. 4C). Nevertheless, a borderline significance of difference was confirmed between the overall S + RT and RT groups (*P* = 0.053, Fig. 4D).

Taken together, the S group experienced the worst OS, while the PFS of the S + RT was significantly better that of the RT group for patients with GTVp ≥30 cm^3^.

### Adverse effects of treatment modalities

The common complications associated with radical surgery were pneumonia (19%) and anastomotic leakage (6.3%; Table 3). Rare complications included wound infection (0.9%), chylothorax (1.7%), respiratory failure (1.1%), anastomotic stenosis (1.4%), and cardiac insufficiency (0.6%). The rates of complications of the S + RT and S groups were similar.

**Table 3:** Surgical complications of the S and S + RT groups

Surgical complications	Total	S	S + RT	χ^2^	*P* value
Pneumonia	67(19.0)	46(19.3)	21(18.4)	0.041	0.839
Anastomotic leakage	22(6.3)	19(8.0)	3(2.6)	2.910	0.088
Wound infection	3(0.9)	3(1.3)	0(0.0)	0.341	0.559
Chylothorax	6(1.7)	5(2.1)	1(0.9)	0.152	0.697
Respiratory failure	4(1.1)	3(1.3)	1(0.9)	0.000	1.000
Anastomotic stenosis	5(1.4)	4(1.7)	1(0.9)	0.013	0.909
Cardiac insufficiency	2(0.6)	1(0.4)	1(0.9)	0.000	1.000

The common side effects of radiotherapy were acute radiation-induced esophagitis (49.7%) and radiation-induced pneumonitis (15.1%); most side effects were complication of grades I -II (Table 4). The rate of acute radiation-induced esophagitis in the S + RT group (64.4%) was markedly lower than that of the RT group (22.0%, *P* < 0.001).

**Table 4:** Radiation toxicity of the S and S + RT groups

Radiation toxicity	Total	RT	S + RT	χ^2^	*P*
Acute radiation pneumonia	50(15.1)	34(15.8)	16(14.1)	0.169	0.681
				2.741	0.433
I	43(13.0)	28(13.0)	15(13.2)		
II	4(1.2)	3(1.4)	1(0.9)		
III	3(0.9)	3(1.4)	0(0.0)		
Acute radiation esophagitis	164(49.7)	139(64.4)	25(22.0)	53.716	<0.001
				57.795	<0.001
I	121(36.7)	102(47.2)	19(16.7)		
II	41(12.4)	36(16.7)	5(4.4)		
III	2(0.6)	1(0.5)	1(0.9)		

## Discussion

To our knowledge, the present study comprised the largest cohort of patients with upper ESCC to investigate the significance of GTVp for prognosis and indication of treatment. This study found that a GTVp of 30 cm^3^ was the optimal cut-off for differences in survival, and GTVp ≥30 cm^3^ was an independent negative risk factor of OS and PFS. For GTVp < 30 cm^3^, no significant survival differences were observed among the 3 treatment groups (S, RT, and S + RT). For patients with GTVp ≥30 cm^3^, the S + RT and RT groups both experienced significantly better OS than did the S group, while the PFS of the S + RT group was superior to that of the RT. In addition, the S + RT group had a significantly lower rate of radiation side effects compared with the RT group. These findings indicate that GTVp was predictive for prognosis and treatment in patients with upper ESCC.

In this study, we firstly confirmed the GTVp-dependent effect on survival, and determined that 30 cm^3^ was the optimal cut-off point using P-spline regression analyses. GTVp was then found to be an independent prognostic factor for OS and PFS. Recently, several studies have reported a close association between survival outcome and tumor volume in EC [19, 20], but the optimal cut-off points of GTVp varied among studies. Créhange et al. [7] initially found that the optimal cut-off point for differences in OS was 100 cm3. Chen et al. [21] reported that 20 cm^3^ and 40 cm^3^ were useful cut-off points for OS differences, based on clinical experience. Using receiver operating characteristic (ROC) curve analysis, Chen et al. [22] identified 39.41 cm^3^ as a cut-off to predict survival differences, while Chen et al. [10] suggested a cut-off of 28 cm^3^. The above postulated cut-off values were based either on clinical experience or ROC analysis, and limited by considering only associations between the continuous variable and survival endpoints. In the present study, multivariate Cox regression analysis using P-splines in smoothHR was conducted to determine the optimal cutoff value, which should be more valid because it is based on an association between the continuous variable and survival rates.

To further verify the prognostic value of GTVp, PSM analysis was employed to calibrate the potential indication bias between patients with GTVp < 30 cm^3^ and those with GTVp ≥30 cm^3^. It was observed that patients with GTVp ≥30 cm^3^ indeed experienced poorer OS and PFS rates. In addition, multivariate Cox proportional hazards regression showed that GTVp (HR [hazard ratio] for OS 1.682; HR for PFS 1.673) was more prognostic than T stage (HR for OS 1.245; HR for PFS 1.214). Therefore, GTVp is an important risk factor that independently influences patient outcomes, outside of clinical TNM stage.

An increasing number of studies have indicated that TNM stage may not be sufficient to determine prognosis, as it does not consider GTV that differs between T or N stage [10, 22]. Larger GTVp is understood to represent greater tumor load, a higher percentage of tumor radioresistant hypoxic cells and clonogenic cells, and greater risk to organs surrounding the tumor, which results in poor survival [21]. Furthermore, GTVp should be included in the staging system as an indicator of patients’ prognosis [9, 10]. Taken together, GTVp showed a powerful prognostic value that may make up for the deficiency of the TNM stage, and should be part of a personalized treatment strategy for upper ESCC.

As GTVp in the present study was markedly different among the S, RT, and S + RT groups, the 3 treatment groups were further stratified by GTVp to explore prognostic effect. For GTVp < 30 cm^3^, there were no significant differences in OS and PFS among the treatments. This may be because of the lighter tumor burden and less tumor infiltration to adjacent anatomical structures, and GTVp < 30 cm^3^ may result in a significantly better prognosis irrespective of the therapeutic methods. It is noteworthy that 215 (54.3%) and 80 (20.2%) patients, respectively, were included in the S and S + RT groups (Supplemental Table 1), which suggests that most of the patients with GTVp < 30 cm^3^ were considered viable candidates for radical resection. Even for the 101 (25.5%) patients who did not undergo surgery, definitive radiotherapy was able to achieve good efficacy, because radioresistant hypoxic and clonogenic tumor cells were negligible when the GTVp is less than 30 cm^3^. Therefore, for patients with GTVp < 30 cm^3^, we suggest that surgery, or radiotherapy plus surgery, is preferred if the tumors are considered resectable, whereas definitive radiotherapy is best if the tumors are non-resectable.

For GTVp ≥30 cm^3^, the S group had the worst 5-year OS rate. The extent of tumor local invasion was larger, and most of these patients (81.4%) were staged as locoregional advanced disease. Thus, surgery may not be sufficient to control tumor development when GTVp is ≥30 cm^3^, and poor OS is the result.

Furthermore, the S + RT group had better 5-year PFS rates than did the RT group. This may be because, first, a more advanced N stage may have been a feature of the RT group. Although the imbalance in clinical characteristics between the RT and S + RT groups was compensated for by PSM, more numbers of positive locoregional nodal metastases may be detected in the RT group if it was given lymph nodal pathological examination. There is surgical data that the rates of positive locoregional nodal metastases in ESCC increase with each T stage, from a low of 31% for T1b, up to 100% for T4 [23].

Secondly, the RT group may have had lower PFS because these patients were more prone to develop cervical lymph node metastases than the S + RT group. It was reported that the most common nodal metastases in upper ESCC are cervical (49.5%) [15]. In addition, patients who received definitive chemoradiotherapy experienced more cervical lymph node recurrences compared with those given radical esophagectomy for ESCC [24], which may be reflected the poorer 5-year PFS rates in the RT group of the present study.

Finally, perhaps the S + RT group had better 5-year PFS rates than the RT because the addition of radiotherapy to radical surgery made loco-regional recurrence more unlikely. Some studies showed that neoadjuvant chemoradiotherapy prior to surgery could result in lower risk of local recurrence and higher disease-free survival in patients with locally advanced ESCC [25, 26]. Consequently, in the present study, the addition of radiotherapy to surgery was associated with preferable survival benefits in patients with GTVp ≥30 cm^3^.

The S + RT group had no more surgical complications than did the S group. Postoperative complications that have been confirmed negative prognostic factors in EC include pneumonia, pyothorax, and chylothorax, and preoperative chemoradiotherapy was even reported to reduce the negative effect of postoperative complications on patient outcome [27]. In the present study, most radiotherapy treatments when given were delivered postoperatively (16.7% preoperatively), and the S + RT group did not have a significant rate of postoperative complications. Furthermore, the S + RT group showed a significantly lower rate of radiation side effects compared with the RT group, especially for acute radiation-induced esophagitis. This may be because the radiation dose delivered during definitive radiotherapy was much higher than that of either the preoperative or postoperative radiotherapy.

Notably, there were some limitations in this study. Although the cohort was large, it was retrospective, with inevitable selection and confounding bias. A prospective, multicenter study is warranted. Furthermore, this study did not consider the GTV of lymph nodes, which may also influence survival [9]. Because the selection of treatment was primarily based on T stage for non-metastatic ESCC as recommended by the NCCN guideline [3], we emphasized the prognostic and predictive effects of GTVp. Yet, the effects of the GTV of lymph nodes for upper ESCC are worthy of investigation. Finally, this analysis did not integrate other independent prognostic factors with GTVp, such as gender, cT stage, and LNM, which may improve the accuracy of prognosis and choice of treatment. Thus, a nomogram to predict the survival prognosis of patients with upper ESCC is needed.

In conclusion, GTVp is prognostic for OS and PFS in patients with upper ESCC. For GTVp < 30 cm^3^, no significant survival differences were observed among the RT, S, and S + RT treatment arms. For ESCC GTVp ≥30 cm^3^, radiotherapy plus surgery was the most effective treatment. These findings may help clinicians strategize individualized treatment for patients with upper ESCC.

## Supplementary Information

**Additional file 1: Table S1.** Clinical characteristics of 568 patients sorted by GTV-p before PSM, and 264 patients after PSM.

**Additional file 2: Table S2.** Clinical characteristics of 306 patients in the RT and S groups before PSM, and 158 patients after PSM for GTV-*p* < 30 cm^3^.

**Additional file 3: Table S3.** Clinical characteristics of 149 patients in the RT and S + RT groups before PSM, and 54 patients after PSM for GTV-*p* ≥ 30 cm^3^.

## Data Availability

The data that support the findings of this study are available from Fujian Medical University Cancer Hospital, but restrictions apply to the availability of these data, which were used under license for the current research, and so are not publicly available. Data are, however, available from the corresponding authors upon reasonable request and with permission of Fujian Medical University Cancer Hospital.

## Abbreviations

AJCC: American Joint Committee on Cancer
cTNM: clinical TNM
EC: Esophageal cancer
ESCC: Esophageal squamous cell carcinoma
GTVp: Primary gross tumor volume
InHR: Indicated hazard ratio
LNM: Lymph node metastasis
NCCN: National Comprehensive Cancer Network
OS: Overall survival
PFS: Progression-free survival
PSM: Propensity score matching
P-splines: Penalized splines
ROC: Receiver operating characteristic
TNM: Tumor-node-metastasis
UICC: Union for International Cancer Control
