# Supplementary material for: Primary gross tumor volume is prognostic and suggests treatment in upper esophageal cancer
Source: BMC Cancer. 2021 Oct 21;21:1130. doi: 10.1186/s12885-021-08838-w (PMC8529770; doi:10.1186/s12885-021-08838-w)
Supplement: Supplementary file 1 — Additional file 1: Table S1. Clinical characteristics of 568 patients sorted by GTV-p before PSM, and 264 patients after PSM. [file 12885_2021_8838_MOESM1_ESM.doc]

|  |  | Pre-PSM |  |  | Post-PSM |  |
| --- | --- | --- | --- | --- | --- | --- |
| Characteristics | GTV- p＜ 30cm^3^ | GTV- p ≥ 30cm^3^ | *P* value | GTV- p ＜ 30cm^3^ | GTV- p ≥ 30cm^3^ | *P* value |
| n(%) | 396（69.7） | 172（30.7） |  | 132(50.0) | 132(50.0) |  |
| Gender |  |  | 0.002 |  |  | 0.045 |
| Male | 257(64.9) | 134（77.9） |  | 84 (63.6) | 99（75.0） |  |
| Female | 139(35.1) | 38（22.1） |  | 48(36.4) | 33（25.0） |  |
| Age (year) |  |  | 0.175 |  |  | 0.806 |
| ＜ 60 | 185（46.7） | 91（52.9） |  | 64（48.5） | 66（50.0） |  |
| ≥ 60 | 211（53.3） | 81（47.1） |  | 68（51.5） | 66（50.0） |  |
| LNM |  |  | ＜ 0.001 |  |  | 0.258 |
| No | 195(49.2) | 57（33.1） |  | 57(43.2) | 48（36.4） |  |
| Yes | 201(50.8) | 115（66.9） |  | 75(56.8) | 84（63.6） |  |
| cT stage |  |  | ＜ 0.001 |  |  | 0.959 |
| T_0-2_ | 115（29.0） | 12（7.0） |  | 13（9.8） | 12（9.1） |  |
| T_3_ | 188（47.5） | 59（34.3） |  | 55（41.7） | 57（43.2） |  |
| T_4_ | 93（23.5） | 101（58.7） |  | 64（48.5） | 63（47.7） |  |
| cN stage |  |  | ＜ 0.001 |  |  | 0.786 |
| N0 | 235（59.3） | 50（29.1） |  | 47（35.6） | 44（33.3） |  |
| N1 | 115（29.0） | 70（40.7） |  | 51（38.6） | 49（37.9） |  |
| N2-3 | 46（11.6） | 52（30.2） |  | 34（25.8） | 39（27.7） |  |
| cTNM stage |  |  | ＜ 0.001 |  |  | 0.962 |
| I-II | 229（57.8） | 32(18.6) |  | 31（23.5） | 32(24.2) |  |
| III | 77（19.4） | 40(23.3) |  | 39（29.5） | 37(28.0) |  |
| IV | 90（22.7） | 100(58.1) |  | 62（47.0） | 63(47.7) |  |
| Tumor length |  |  | ＜ 0.001 |  |  | 0.036 |
| ≤ 5cm | 307（89） | 56（32.6） |  | 73（55.3） | 56（42.4） |  |
| ＞ 5cm | 89（22.5） | 116（67.4） |  | 59（44.7） | 76（57.6） |  |
| Treatment mode |  |  | ＜ 0.001 |  |  | 0.461 |
| S | 215（54.3） | 23（13.4） |  | 27（20.5） | 23（17.4） |  |
| RT | 101（25.5） | 115（66.9） |  | 70（53.0） | 80（60.6） |  |
| S+RT | 80（20.2） | 34（19.8） |  | 35（26.5） | 29（22.0） |  |

Table S1. Clinical characteristics of 568 patients sorted by GTV-p before PSM, and 264 patients after PSM.
