# Supplementary material for: Primary gross tumor volume is prognostic and suggests treatment in upper esophageal cancer
Source: BMC Cancer. 2021 Oct 21;21:1130. doi: 10.1186/s12885-021-08838-w (PMC8529770; doi:10.1186/s12885-021-08838-w)
Supplement: Supplementary file 2 — Additional file 2: Table S2. Clinical characteristics of 306 patients in the RT and S groups before PSM, and 158 patients after PSM for GTV-p < 30 cm3. [file 12885_2021_8838_MOESM2_ESM.doc]

|  | Pre-PSM | |  | Post-PSM | |  |
| --- | --- | --- | --- | --- | --- | --- |
| Characteristics | R | S | *P* value | R | S | *P* value |
| n(%) | 101（32.0） | 215（68.0） |  | 79（50.0） | 79（50.0） |  |
| Gender |  |  | 0.402 |  |  | 0.747 |
| Male | 57 (56.4) | 132 (61.4) |  | 47 (59.5) | 45（57.0） |  |
| Female | 44 (43.6) | 83 (38.6) |  | 32 (40.5) | 34（43.0） |  |
| Age (year) |  |  | 0.021 |  |  | 0.423 |
| ＜ 60 | 34（33.7） | 102（47.4） |  | 32（40.5） | 37（46.8） |  |
| ≥ 60 | 67（66.3） | 113（52.6） |  | 47（59.5） | 42（53.2） |  |
| LNM |  |  | 0.001 |  |  | 0.426 |
| No | 40（39.6） | 127（59.1） |  | 40（50.6） | 35（44.3） |  |
| Yes | 61（60.4） | 88（40.9） |  | 39（49.4） | 44（55.7） |  |
| cT stage |  |  | ＜ 0.001 |  |  | 0.251 |
| T0-2 | 14（13.9） | 80（37.2） |  | 14（17.7） | 17（21.5） |  |
| T3 | 32（31.7） | 109（50.7） |  | 32（40.5） | 39（49.4） |  |
| T4 | 55（54.5） | 26（12.1） |  | 33（41.8） | 23（29.1） |  |
| cN stage |  |  | ＜ 0.001 |  |  | 0.837 |
| N0 | 40（39.6） | 145（67.4） |  | 40（50.6） | 37（46.8） |  |
| N1 | 40（39.6） | 54（25.1） |  | 31（39.2） | 32（40.5） |  |
| N2-3 | 21（20.8） | 16（7.4） |  | 8（10.1） | 10（12.7） |  |
| cTNM stage |  |  | ＜ 0.001 |  |  | 0.362 |
| I-II | 33（32.7） | 152（70.7） |  | 33（41.8） | 36(45.6) |  |
| III | 18（17.8） | 36（16.7） |  | 15（19.0） | 20(25.3) |  |
| IV | 50（49.5） | 27（12.6） |  | 31（39.2） | 23(29.1) |  |
| Tumor length |  |  | 0.287 |  |  | 0.714 |
| ≤ 5cm | 77（76.2） | 175（81.4） |  | 60（75.9） | 58（73.4） |  |
| ＞ 5cm | 24（23.8） | 40（18.6） |  | 19（24.1） | 21（26.6） |  |

Table S2. Clinical characteristics of 306 patients in the RT and S groups before PSM, and 158 patients after PSM for GTV-p < 30 cm^3^.
