# Supplementary material for: Primary gross tumor volume is prognostic and suggests treatment in upper esophageal cancer
Source: BMC Cancer. 2021 Oct 21;21:1130. doi: 10.1186/s12885-021-08838-w (PMC8529770; doi:10.1186/s12885-021-08838-w)
Supplement: Supplementary file 3 — Additional file 3: Table S3. Clinical characteristics of 149 patients in the RT and S + RT groups before PSM, and 54 patients after PSM for GTV-p ≥ 30 cm3. [file 12885_2021_8838_MOESM3_ESM.docx]

Supplemental Table 3. Clinical characteristics of 149 patients in the RT and S+RT groups before PSM, and 54 patients after PSM for GTV-p ≥ 30 cm3.

|  |  | Pre-PSM |  |  | Post-PSM |  |
| --- | --- | --- | --- | --- | --- | --- |
| Characteristics | R | R+S | *P* value | R | R+S | *P* value |
| n(%) | 115（77.2） | 34（22.8） |  | 27（50.0） | 27（50.0） |  |
| Gender |  |  | 0.360 |  |  | 0.484 |
| Male | 86 (74.8) | 28（82.4） |  | 23（85.2） | 21（77.8） |  |
| Female | 29 （25.2) | 6（17.6） |  | 4（14.8） | 6（22.2） |  |
| Age (year) |  |  | 0.129 |  |  | 0.154 |
| ＜60 | 54（47.0） | 21（61.8） |  | 20（74.1） | 15（55.6） |  |
| ≥60 | 61（53.0） | 13（38.2） |  | 7（25.9） | 12（44.4） |  |
| LNM |  |  | 0.000 |  |  | 0.276 |
| No | 27（23.5） | 22（64.7） |  | 11（12.5） | 15（31.3） |  |
| Yes | 88（76.5） | 12（35.3） |  | 16（87.5） | 12（68.8） |  |
| cT stage |  |  | 0.037 |  |  | 0.845 |
| T0-2 | 6（5.2） | 5（14.7） |  | 3（11.1） | 3（11.1） |  |
| T3 | 33（28.7） | 14（41.2） |  | 12（44.4） | 10（37.0） |  |
| T4 | 76（66.1） | 15（44.1） |  | 12（44.4） | 14（51.9） |  |
| cN stage |  |  | 0.059 |  |  | 0.824 |
| N0 | 27（23.5） | 15（44.1） |  | 11（40.7） | 12（44.4） |  |
| N1 | 51（44.3） | 12（35.3） |  | 8（29.6） | 9（33.3） |  |
| N2-3 | 37（32.2） | 7（20.6） |  | 8（29.6） | 6（22.2） |  |
| cTNM stage |  |  | 0.063 |  |  | 0.734 |
| I-II | 17（14.8） | 10(29.4) |  | 8（29.6） | 5(22.2) |  |
| III | 23（20.0） | 9(26.5) |  | 5（18.5） | 7(25.9) |  |
| IV | 75（65.2） | 15(44.1) |  | 14（51.9） | 14(51.9) |  |
| Tumor length |  |  | 0.278 |  |  | 0.214 |
| ≤ 5cm | 42（36.5） | 9（9） |  | 5（18.5） | 9（33.3） |  |
| ＞ 5cm | 73（63.5） | 25（67.4） |  | 22（100.0） | 18（66.7） |  |
